# Abnormalities in Automatic Processing of Illness-Related Stimuli in Self-Rated Alexithymia

**DOI:** 10.1371/journal.pone.0129905

**Published:** 2015-06-19

**Authors:** Laura Brandt, Nina M. Pintzinger, Ulrich S. Tran

**Affiliations:** 1 Center for Public Health, Medical University of Vienna, Kinderspitalgasse 15, 1090 Vienna, Austria; 2 Faculty of Psychology, University of Vienna, Liebiggasse 5, 1010 Vienna, Austria; Radboud University Nijmegen, NETHERLANDS

## Abstract

**Aim:**

To investigate abnormalities in automatic information processing related to self- and observer-rated alexithymia, especially with regard to somatization, controlling for confounding variables such as depression and affect.

**Sample:**

89 healthy subjects (60% female), aged 19–71 years (M = 32.1). 58 subjects were additionally rated by an observer.

**Measures:**

Alexithymia (self-rating: TAS-20, observer rating: OAS); automatic information processing (priming task including verbal [illness-related, negative, positive, neutral] and facial [negative, positive, neutral] stimuli); somatoform symptoms (SOMS-7T); confounders: depression (BDI), affect (PANAS).

**Results:**

Higher self-reported alexithymia scores were associated with lower reaction times for negative (r = .19, p < .10) and positive (r = .26, p < .05) verbal primes when the target was illness-related. Self-reported alexithymia was correlated with number (r = .42, p < .01) and intensity of current somatoform symptoms (r = .36, p < .01), but unrelated to observer-rated alexithymia (r = .11, p = .42).

**Discussion:**

Results indicate a faster allocation of attentional resources away from task-irrelevant information towards illness-related stimuli in alexithymia. Considering the close relationship between alexithymia and somatization, these findings are compatible with the theoretical view that alexithymics focus strongly on bodily sensations of emotional arousal. A single observer rating (OAS) does not seem to be an adequate alexithymia-measure in community samples.

## Introduction

Historically, Sifneos (1972) defined alexithymia as a categorical clinical construct, manifesting itself in an inability to perceive and describe emotions sufficiently [1]. Competing definitions, mainly based on clinical observations [2], were replaced by the current concept of alexithymia, based on operational diagnostics, as a dimensional personality trait with affective and cognitive characteristics, including (1) difficulty identifying feelings and distinguishing them from the bodily sensations of emotional arousal; (2) difficulty describing feelings; (3) paucity of fantasies; and (4) an externally orientated cognitive style [3]. Absolute and relative stability of alexithymia in the general population were shown to be high, even in an 11-year follow-up [4].

Originally, the alexithymia concept was seen as an explanatory model for psychosomatic disorders [5]. While studies investigating the relationship between alexithymia and somatoform disorders as a dichotomous variable (diagnosis yes/no) show significantly elevated alexithymia scores in patients compared to healthy controls [6–8], recent studies indicate that alexithymia is not specific to patients with psychosomatic disorders [9]. High levels of alexithymia are also linked to increased rates of eating disorders, depression, anxiety disorders, certain personality disorders and addiction [10]. However, positive associations between alexithymia and higher somatic symptom reporting were found in the general population independent of somatic disorders, depression, anxiety, and socio-demographic variables, suggesting a close correlation between somatoform disorders and alexithymia [11].

Alexithymia has been linked to problems in information processing of emotional stimuli. Cognition and processing of exteroceptive emotional stimuli are based on two distinct but complementary processes–automatic and controlled processing [12]. A pivotal question with regard to alexithymia relates to the examination, which part of information processing is affected. One study found that subjects high in alexithymia are able to perceive emotions, and that they even seem to perceive negative emotions better than non-alexithymics [13]. However, alexithymics show rudimental affective and undifferentiated cognitive schemata in the processing of emotions, indicating that high alexithymics require more cognitive resources for processing emotional information [13,14].

On a controlled level of information processing, alexithymia seems to involve an impairment in encoding and transforming emotional information, especially in faces, but also in verbal stimuli [15–17], A recent study also found a strong association between recognition of verbally expressed emotions and alexithymia scores in adults with autism spectrum disorder (ASD) and non-ASD controls [18]. These impairments become more severe under time pressure, suggesting an efficiency deficit emerging during increased demand on processing capacity, such as stressful conditions, rather than a general impairment in the capacity for emotion information processing in alexithymia [19,20].

However, only few experimental studies, mainly using stroop-tasks, examined the automatic processing of emotional information in alexithymia. Müller et al. [21] reported among inpatients with psychosomatic disorders a diminished emotional bias (i.e., faster color-naming of emotional stimuli words) in observer-rated alexithymics compared to non-alexithymics for negative and bodily symptom words. No difference was found in the explicit rating of the emotional valence of words. This led to the conclusion that alexithymic patients concentrate less attentional resources to negative information. However, no association was found for self-rated alexithymia.

Even fewer studies examined Information processing in alexithymia with sequential affective priming-tasks. In the affective priming paradigm, effects of the emotional valence of a briefly presented stimulus, the prime, are investigated with regard to the processing of a subsequent stimulus, the target. If prime and target are of similar valence, facilitation-effects are expected, i.e., faster reaction times (RTs) as compared to control trials with neutral stimuli. If prime and target are of different valence, inhibition-effects are observed, i.e., slower RTs in comparison to control trials.

Using a sequential priming-task, Suslow [22] found a positive, but only small association between self-rated alexithymia and facilitation-effects for congruent positive word pairs. Using verbal and facial stimuli, Suslow, Junghanns, Donges, and Arolt [23] found facilitation-effects for congruent negative word-word pairs in subjects with high self-rated alexithymia, but not for facial stimuli. This was interpreted as a reduced processing engagement towards negative word stimuli at an automatic level. However, results were contradictory regarding the valence of stimuli, i.e., associations between alexithymia and facilitation-effects were observed in Suslow [22] only for positive stimuli, but in Suslow et al. [23] only for negative stimuli.

In a multi-study paper, Vermeulen, Luminet and Corneille [24] reported an inverse relationship between alexithymia and facilitation-effects with angry face–negative word pairs, which could be replicated in a second study. Yet, in a consecutive third trial, using verbal-facial stimuli pairs, no moderating effect of alexithymia was found, suggesting that previous results could not be simply explained by transcoding limitations between verbal and non-verbal information, but rather by alexithymia being related to a difficulty in automatically processing high-arousal emotional information.

Even though these studies provided important insights into the role of alexithymia in the automatic processing of affective information, their generalizability and validity appear limited. First, samples were partly rather small (e.g., *N* = 32 in Suslow [22]; *N* = 45 in Müller et al. [21]). Second, some studies [22] did not include assessments of mood in their design, or did not control for mood in analysis [23]. Finally, Suslow [22] provided no information on the selection of verbal stimuli and Suslow et al. [23] used only word length as a selection criterion. Thus, aspects other than valence (e.g., familiarity) could have caused contradictory results. Most importantly, however, primes were presented for 200ms with a stimulus onset asynchrony (SOA) of 300ms [23]. With presentation times that long, it is not guaranteed that stimuli were processed on an automatic level, since SOA needs to be below 300ms to rule out controlled processing [25].

### The present study

The aim of the present study was to investigate abnormalities in automatic information processing of affective verbal and facial information in relation to self- and observer-rated alexithymia in a representative community sample with a sequential affective priming-task. We hypothesized that subjects with high alexithymia scores would allocate less attentional resources towards task-irrelevant information if negative or illness-related targets were presented, resulting in increased facilitation-effects for these conditions [21,23,24].

A community sample was chosen, since alexithymia is not only a vulnerability factor for psychiatric disorders [9], but leads to an overall significant decrease in quality of life [26]. Franz et al. [27] reported a prevalence rate of 10% for alexithymia in the general population. Previous studies relied on patient [21,22] or student samples [23,24], hardly representative for the general population. With the exception of Müller et al. [21], previous studies used only self-ratings for measuring alexithymia. Investigating a sufficiently large, representative community sample and including observer ratings may thus increase the generalizability of results and validity of assessment. Because of the association of alexithymia with somatization, somatization was also controlled for in this study, as were positive and negative affect, and depressive symptoms [28,29]. The priming task was designed to include verbal as well as facial stimuli. The valence (positive, negative, neutral) of the verbal stimuli was validated in a prior pilot study and facial stimuli were selected from the elaborately validated FACES database stimuli [30]. Presentation times were chosen to ensure automatic information processing and comparability with previous studies [21,24].

## Method

### Ethics statement

The study was approved by the department of psychology and performed in accordance with the ethical standards of the 1964 Declaration of Helsinki and Austrian ethical regulations for clinical research. All participants gave written informed consent prior to inclusion in the study.

According to national (Austrian) and European (EU) law, approval by an ethics committee was not necessary because the study did not involve patients, was non-invasive, and participation was voluntary and anonymous. There is no further institutional review board (IRB) at the University of Vienna, where the research was conducted. Hence, no IRB approval was necessary.

### Subjects

Previous studies [21,24] suggested a medium effect size regarding the association of alexithymia with the targeted variables. On this basis, a sample size of *N* = 70 was determined sufficient, calculated using G*Power 3 [31]. Participants were sampled to represent the German and Austrian general population, regarding gender distribution, age and education [32,33]. Exclusion criteria were insufficient German language skills, dyslexia, insufficient visual acuity, and a medical history, including brain injury, mental retardation or reduced verbal intelligence (assessed verbally prior to the test procedure). Participants were recruited using a word-of-mouth strategy. Participation in the study was completely voluntary and anonymous. The final sample consisted of 89 native German speakers (54 women) with a mean age of 32.1 years (*SD* = 12.9), ages ranging from 19 to 71 years. 23.6% of the participants had lower secondary education, 48.3% had upper secondary education, and 28.1% had graduated in higher education. Self-rated alexithymia scores (TAS-20; see below) ranged from 25 to 66 (*M* = 41.8, *SD* = 8.9; difficulty identifying feelings [DIF]: *M* = 13.56, *SD* = 4.2; difficulty describing feelings [DDF]: *M* = 11.09, *SD* = 3.6; externally oriented thinking [EOT]: *M* = 17.10, *SD* = 4.2) and were normally distributed.

58 of the subjects were rated by an observer. Observers knew the rated subjects between 1.5 and 45 years (*M* = 18.6, *SD* = 10.3). Of observers, 13.8% were parents, 17.2% siblings, 46.6% partners, and 19% children of the rated subjects. Observer-rated subjects were older (*M* = 34.7, *SD* = 14.3 vs. *M* = 27.2, *SD* = 7.5, *p* = .01) than subjects without observer rating. However, TAS-20 scores did not differ significantly between observer (*M* = 42, *SD* = 8.7) and not-observer rated participants (*M* = 41, *SD* = 9.4, *p* = .52).

### Measures

#### Toronto Alexithymia Scale 20 (TAS-20)

The German 20-item TAS was used for the assessment of self-reported alexithymia [34]. The questionnaire contains scales on the difficulty identifying feelings (DIF), the difficulty describing feelings (DDF), and externally oriented thinking (EOT), rated on a 5-point Likert scale ranging from 1 (*strongly disagree*) to 5 (*strongly agree*). TAS-20 total scores range from 20 to 100, with high scores indicating high alexithymia. In the present study, percentiles 33 and 66 were used to form low and high alexithymia groups as suggested by Franz et al. [27]. The TAS-20 was chosen for this study to ensure comparability of results with relevant studies examining the automatic processing of emotional information in alexithymia [21–24], of which all have used the TAS- as well. Other scales, like the Bermond-Vorst Alexithymia Questionnaire [35], may provide a more elaborated and comprehensive operationalization of alexithymia than the TAS-20. However, the convergent validity of the Observer Alexithymia Scale (see below), which was also used in this study, was previously just provided with regards to the TAS-20 [36].

#### Observer Alexithymia Scale (OAS)

The OAS is an instrument for measuring observer-rated alexithymia [37]. In the present study, the unpublished German form was used, which was provided by Mark Haviland and Wolfgang Sitte. Haviland et al. [37] suggested a 5-factor structure: (1) distant (poor interpersonal skills and relationships); (2) uninsightful (poor stress tolerance, insight, and self-understanding); (3) somatizing (health worries and physical problems); (4) humorless (uninteresting and boring); and (5) rigid (excessive self-control). This highlights a rather broad understanding of the alexithymia concept in the OAS. In the present study, total OAS scores were used.

#### Control Variables

The positive and negative affect scale (PANAS) was used to assess positive and negative affect [38]. For the assessment of self-rated depressive symptoms, the Beck Depression Inventory was used (BDI) [39]. The 7-day version of the Screening for Somatoform Symptoms (SOMS) was used to assess the quantity and intensity of somatoform symptoms in the last 7 days [40].

#### Priming Task

Verbal stimuli consisted of 12 positive (positive feelings; e.g., happiness), 12 negative (negative feelings; e.g., sadness), 12 neutral (professions; e.g., interpreter) and 12 illness-related words (negative words related to bodily symptoms; e.g., dizziness). Verbal stimuli were taken from Müller et al. [21]. The inclusion of bodily symptom words allowed assessing sensitivity to bodily symptoms in high alexithymic persons [3]. Word stimuli in Müller et al. [21] were controlled for frequency of use, familiarity, length, and number of syllables across categories. For the present study, the valence (positive, negative, neutral) of the stimuli was validated in a prior pilot study, using an independent sample of 39 healthy subjects (18 to 60 years of age, 21 women). This validation sample was comparable to the study sample with respect to the distribution of age, gender, and level of education. Subjects rated all 36 words on a 7-point scale regarding their valence (1 = *extremely negative* to 7 = *extremely positive*). The valence of all words but “Kribbeln” (tingling; assumed negative valence, but rated positive here) could be confirmed. “Kribbeln” was replaced by “Juckreiz” (itching).

Facial stimuli were taken from the FACES database of the Max Planck Institute for Human Development; they show high interrater agreement regarding ratings of facial expression as well as high percentages regarding identifiability of expressions [30]. Faces of seven middle aged posers were selected, showing angry, happy, and neutral expressions. Faces of three posers were used in practice trials and faces of four posers (2 women, 2 men) were used in the actual task.

**Fig 1:**
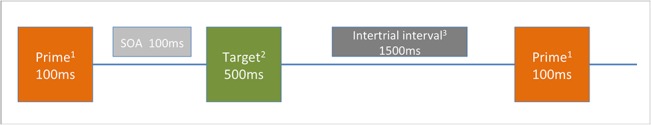
Presentation times of the priming task elements. ^1^Primes were verbal stimuli (positive, negative, neutral, or illness-related) or facial stimuli (angry, happy, or neutral); ^2^Targets were verbal stimuli (positive, negative, or illness-related) or facial stimuli (angry or happy); ^3^During the intertrial interval a fixation cross was presented; SOA = stimulus onset asynchrony.

The priming task was programmed with E-Prime 2.0 Runtime. The task consisted of four blocks: 1. verbal prime–facial target, 2. verbal prime–verbal target, 3. facial prime–facial target, and 4. facial prime–verbal target. In each block each stimulus appeared at least two times. The presentation sequence was randomized, but the same two stimuli were never combined and targets were never neutral. Facial stimuli were presented in a size of 1280x1024 pixels, centric on a light grey background. Verbal stimuli were presented in typesize 18, typeface Courier New, colored black, also centric on a light grey background. Stimuli were presented on a Sony Vaio Laptop (Model PCG-7M1M) with a 15.4 inch monitor. Presentation times were adopted from Vermeulen et al. [24] and were identical for all four blocks of the task (see Fig 1). Response latencies were recorded by pressing one of two response keys, F for negative and J for positive, respectively, on a QWERTZ keyboard. Pressing F in case of a positive target, and J in case of a negative target, was recorded as a rating error.

### Procedure

Subjects were tested in single sessions in an undisturbed and quiet room. Subjects should evaluate each target as positive or negative as quickly and accurately as possible (i.e., explicit rating of emotions). All subjects completed six practice trials prior to the four blocks of the task to become familiar with the procedure. After finishing the priming task, participants filled out the questionnaire. At the end of the trial, subjects were asked if they agreed to an assessment by others. Observer rating was also voluntary and anonymous. Choice of rater (limited to parents, children of age, siblings or partner, if the relationship lasted at least a year) was up to the participants. If participants agreed to be externally assessed, a questionnaire including the OAS was send to the rater via email.

### Analysis

Correlational analyses were conducted with Pearson correlations, covariates were controlled for with partial correlations. Between-groups analyses were conducted with independent *t*-tests and analysis of covariance (ANCOVA). Alpha was set to *p* < .05, *p*-values between .05 and .15 were interpreted as statistical trends. Effect sizes are reported for significant results; for ANCOVA, effect sizes of η^2^ ≥ 0.02 signified small, η^2^ ≥ 0.13 medium, and η^2^ ≥ .26 large effects. For pairwise contrasts, effect sizes (Cohen’s *d*) of *d* ≥ 0.20 signified small, *d* ≥ 0.50 medium, and *d* ≥ .80 large effects.

Only valid trials in the priming task (i.e., answers agreeing with stimulus valence; e.g., negative for angry faces) were included in analysis, resulting in 82% valid data. Invalid trials were recorded separately. Outliers in the RT data were identified on a group basis, using the 95.5th percentile as a cut-off, which is in accordance with recommendations for RT measures [41].

## Results

There was no significant gender difference in self-reported alexithymia (TAS-20), *t*(87) = 0.19, *p* = .854, and no significant correlation between self-reported alexithymia and education, *r* = -.19, *p* = .086, or age, *r* = -.13, *p* = .234. Only two participants exceeded a TAS-20 score cut-off of 61 [42]. Percentile 33 lay at a TAS-20 score of 37, percentile 66 at 44 [27]. Of the sample, 33.7% were classified as low alexithymic, 25.9% as medium alexithymic, and 40.4% as high alexithymic.

Negative affect correlated positively, *r* = .41, and positive affect negatively, *r* = -.39, with self-reported alexithymia (*p*s < .001). Self-reported alexithymia and depression also correlated, *r* = .39, *p* < .001. Therefore, all three variables were included as confounders in further analysis. Number of somatoform complaints, *r* = .51, *p* < .001, and intensity of complaints, *r* = .49, *p* < .001, also correlated with self-reported alexithymia.

Priming effects in terms of facilitation and inhibition scores were calculated for the whole sample (Table 1). Expected priming-effects were overall more distinct for the congruent than the incongruent conditions; except for block 1 (verbal prime–facial target) where the expected priming-effects were more distinct for the incongruent conditions. RTs for facial targets (*M* = 595.70 ms, *SD* = 12.03) were significantly lower compared to verbal targets (*M* = 705.37 ms, *SD* = 12.95), *F*(1, 88) = 74.61, *p* < .001, η^2^ = .46. Furthermore, RTs for congruent conditions (*M* = 645.86 ms, *SD* = 11.07) were lower compared to incongruent conditions (*M* = 655.21 ms, *SD* = 10.70), *F*(1, 88) = 8.07, *p* = .006, η^2^ = .08.

**Table 1:** Means and Standard Deviations of Facilitation and Inhibition scores (in ms).

Valence of prime	Facilitation	Inhibition
*Block 1 (verbal prime–facial target)*		
Illness-related	-4.9 (56.3)	**-10.7 (57.0)**
Negative	**6.4 (59.8)**	**-17.1 (54.7)**
Positive	**3.5 (59.7)**	**-7.3 (53.0)**
*Block 2 (verbal prime–verbal target)*		
Illness-related	**21.3 (64.0)**	1.9 (57.0)
Illness-related–Negative	**1.3 (61.0)**	NA
Negative	**5.4 (60.0)**	**-0.1 (68.5)**
Negative–Negative	**13.5 (64.1)**	NA
Positive	**7.1 (76.8)**	11.0 (63.5)
Positive–Negative	NA	4.9 (57.2)
*Block 3 (facial prime–facial target)*		
Negative	**6.5 (45.1)**	2.2 (46.2)
Positive	**13.8 (50.0)**	**-3.5 (49.6)**
*Block 4 (facial prime–verbal target)*		
Negative	**1.6 (52.8)**	0.3 (65.1)
Negative–Negative	-6.0 (56.8)	NA
Positive	**10.4 (62.5)**	**-5.6 (53.1)**
Positive–Negative	NA	**-6.4 (57.2)**

*Note*. Facilitation and inhibition scores were calculated by subtracting the means of the congruent conditions (e.g., positive prime–positive target) and the incongruent conditions (e.g., positive prime–negative target), respectively, from the means of the neutral conditions (e.g., neutral prime–positive target). In case of a facilitation score, this value is positive if the affective prime facilitated a faster processing of the congruent target compared to the neutral condition. In case of an inhibition score, this value is negative if the affective prime inhibited the processing of the incongruent target compared to the neutral condition. Numbers are bold if the value is in the expected direction. NA = not applicable.

Self-reported alexithymia did not correlate with observer-rated alexithymia (OAS), *r* = .11, *p* = .42. Furthermore, observer-rated alexithymia was not significantly related to any of the inhibition and facilitation scores (Table 2). Therefore, observer ratings were not included in further analysis.

**Table 2:** Correlations of TAS-20 and OAS Scores with Facilitation and Inhibition scores.

	TAS-20		OAS	
Valence of prime	Facilitation	Inhibition	Facilitation	Inhibition
*Block 1 (verbal prime–facial target)*				
Illness-related	-.25** (-.16)	-.11 (-.07)	-.15	.22
Negative	-.11 (-.12)	-.04 (-.09)	-.16	.24
Positive	-.10 (-.11)	-.07 (-.10)	.17	-.20
*Block 2 (verbal prime–verbal target)*				
Illness-related	.10 (.13) ^a^	.06 (.13)	.18	.12
Illness-related–Negative	-.12 (-.15)	NA	-.06	NA
Negative	.24* (.19^+^)^a^	-.13 (-.08)	-.09	-.01
Negative–Negative	.28** (.16)	NA	-.10	NA
Positive	-.13 (-.08)	.21* (.26*) ^a^	-.09	.02
Positive–Negative	NA	.15 (.06)	NA	.04
*Block 3 (facial prime–facial target)*				
Negative	.08 (.15)	-.02 (.00)	.01	.16
Positive	.06 (.09)	-.04 (.05)	.08	-.07
*Block 4 (facial prime–verbal target)*				
Negative	.08 (.05) ^a^	-.02 (-.02)	.18	-.02
Negative–Negative	.03 (-.02)	NA	-.01	NA
Positive	.06 (.11)	.17 (.11) ^a^	.05	.08
Positive–Negative	NA	-.13 (-.16)	NA	.03

*Note*. For TAS-20 scores, numbers in parentheses are partial correlations, controlling for depression, and positive and negative affect. NA = not applicable.

*p < .05,

**p < .001,

^+^ *p* < .10,

^a^ targets were illness-related.

Only within conditions “negative verbal prime–illness-related target” and “positive verbal prime–illness-related target” significant correlations with self-reported alexithymia, respectively a statistical trend after controlling for confounders, were observed (Table 2). Correcting for multiple testing (Bonferroni method), all associations lost their nominal significance.

To evaluate whether the effect of alexithymia on automatic processing of affective information could be attributed to a specific facet of alexithymia, correlations were calculated between each scale of the TAS-20 and facilitation (“negative verbal prime–illness-related target”) and inhibition scores (“positive verbal prime–illness-related target”), respectively. Only DIF scale scores and facilitation scores correlated significantly, *r* = .23, *p* = .03.

**Fig 2:**
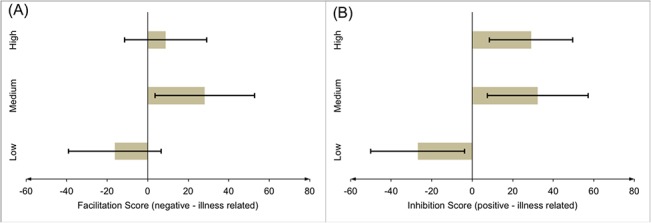
Effects of alexithymia on automatic processing of illness-related information. (A) Estimated marginal means (ANCOVA) of alexithymia groups (high vs. medium vs. low) of facilitation scores under the priming-condition “negative verbal prime–illness related target”, controlling for positive and negative affect and depressive symptoms. (B) Estimated marginal means (ANCOVA) of alexithymia groups (high vs. medium vs. low) of inhibition scores under the priming-condition “positive verbal prime–illness related target”, controlling for positive and negative affect and depressive symptoms. *Note*. With regard to Fig 2(A), it has to be noted that error bars of marginal means overlapped between groups. This does not contradict the results of the statistical tests regarding group mean differences. Whereas error bars in Figs 2(A) and (B) are based on individual within-group estimates of variation, the pairwise statistical tests are based on a more reliable overall estimate of within-group variation, simultaneously using data from all three groups.

ANCOVAs of facilitation and inhibition scores of the priming-conditions “negative verbal prime–illness-related target” and “positive verbal prime–illness-related target” as dependent variables, self-reported alexithymia (high vs. medium vs. low) as independent variable, and positive and negative affect and depressive symptoms as covariates, revealed a medium overall effect of alexithymia on facilitation-scores, *F*(2, 83) = 3.34, *p* = .040, η^2^ = .07, with a difference of medium effect size between low alexithymics (*M* = -16.23, *SE* = 11.5) and medium alexithymics (*M* = 28.15, *SE* = 12.4), *t*(51) = 2.58, *p* = .012, *d* = 0.71, and a difference of small-to-medium effect size, approaching statistical significance, between low and high alexithymics (*M* = 8.87, *SE* = 10.2), *t*(64) = 1.55, *p* = .126, *d* = 0.38 (Fig 2(A)). In inhibition-scores, alexithymia had an overall large effect, *F*(2, 83) = 7.53, *p* = .001, η^2^ = .15. Simple contrasts revealed a significant difference between low (*M* = -26.95, *SE* = 11.65) and medium alexithymics (*M* = 32.40, *SE* = 12.5), *t*(51) = 3.41, *p* = .001, *d* = 0.94, as well as between low and high alexithymics (*M* = 29.05, *SE* = 10.3), *t*(64) = 3.41, *p* = .001, *d* = 0.84 (Fig 2(B)).

There was also a statistical trend regarding the correlation between self-reported alexithymia and rating errors, *r* = .20, *p* = .061. Subjects with higher self-rated alexithymia made more errors than subjects with lower self-rated alexithymia. There was no association between facilitation scores and number of somatoform complaints, *r* = .14, *p* = .204, and intensity, *r* = .11, *p* = .291, and inhibition scores and number of complaints, *r* = .10, *p* = .331, and intensity, *r* = .07, *p* = .538.

## Discussion

The aim of the present study was to assess alexithymia-specific abnormalities in automatic processing of affective verbal and facial information in a representative sample of the German and Austrian general population, under consideration of current somatoform symptoms. Results indicate a specific sensibility of high alexithymics for illness-related stimuli. In line with our hypothesis, self-reported alexithymia was specifically associated with scores in the priming conditions “negative verbal prime–illness-related target” and “positive verbal prime–illness-related target”. With regard to verbal stimuli, high alexithymics showed less allocation of attentional resources towards task-irrelevant information when targets were illness-related; this was not only true for negative (increased facilitation scores compared to low alexithymics), but also for positive primes (diminished inhibition scores compared to low alexithymics). Müller et al. [21] also found a diminished emotional bias in observer-rated alexithymics compared to non-alexithymics for negative and bodily symptom words. However, in contrast to our results, this was found for observer-, but not self-rated alexithymia.

Previous studies provided contradicting results regarding the emotional valence of stimuli. Apart from using a less complex task, Suslow [22] and Suslow et al. [23] did not control for confounding variables, that were found important correlates of alexithymia in the present study, and did not include illness-related stimuli (with the exception of Müller et al. [21]), which might have prompted these ambiguous results. Our results show highly significant correlations of self-reported alexithymia with facilitation scores for negative targets (“illness-related prime–negative facial target”; “negative verbal prime–negative verbal target”). However, these correlations lost their significance after controlling for confounding variables, suggesting that findings by Suslow et al. [23] regarding a reduced processing engagement towards negative stimuli might have been mediated by positive and negative affect, and depression. The positive association of alexithymia and facilitation effects for congruent positive prime-target pairs found by Suslow [22] is in conflict with results from other relevant studies [21,23,24] and could not be replicated.

In the present study, persons scoring high on the difficulties identifying feelings (DIF) dimension of alexithymia showed increased facilitation scores for illness-related targets, which is in accordance with previous findings [43]. It has been shown that the TAS-20 DIF scale is the strongest common link between alexithymia and somatization [11], and high scores in difficulty identifying feelings are highly predictive of a broad range of state levels of psychopathology, particularly somatization [44].

In addition, the close correlation between self-reported alexithymia and number and intensity of somatoform symptoms in the present study underlines that there are alexithymia-specific effects regarding bodily symptoms [3]. The SOMS includes, besides explicit illness-related symptoms (e.g., diarrhea), symptoms that can be bodily sensations of emotional arousal as well as signs of illness (e.g., tingeling or palpitation). This suggests that the relation of alexithymia with psychosomatic disorders [11] may be explained by a stronger focus on bodily sensations and their misinterpretation as signs of illness in alexithymia.

Impaired processing of facial stimuli [24] in alexithymia could not be replicated in our study. However, RTs were generally very low with regard to facial targets, compared to verbal targets, regardless of self-reported alexithymia. The elaborately validated FACES database stimuli [30] could have included very easily identifiable expressions, even for high alexithymics. Furthermore, a systematic review [45] pointed out that alexithymia is associated with deficits in labelling emotional facial expressions among patients with clinical disorders, but that depression and anxiety partially account for these decoding deficits. This interpretation is also supported by the fact that the correlation between alexithymia and the facilitation scores for negative targets in the present study lost their significance after controlling for cofounders.

However, high alexithymics showed higher error rates in rating emotions for both facial and verbal stimuli (cf. [21]). Under consideration of our study procedure (subjects were asked to evaluate targets as quickly and accurately as possible), this result is in line with the hypothesis that alexithymia is not characterized by a general impairment in information processing of affective information (i.e., alexithymics are able to correctly appraise the emotional valence of affective information [21]), but a deficit in efficiency in certain contexts as time pressure [19,20].

The results indicate that the stimuli selection and the priming task elicited measurable priming effects, while some effects were moderated by alexithymia; e.g., no inhibition of responses to verbal illness-related targets after positive verbal primes was found for the whole sample. However, subsequent analysis showed the expected inhibition effect of positive verbal primes for verbal illness-related targets for low-alexithymics, while medium and high alexithymics did not show this inhibition of response. Overall, priming-effects were mostly larger than reported in prior studies (e.g., Suslow [22]: 1–5ms).

No correlation between self- and observer-rated alexithymia could be observed in this study. This may be explained by at least three possible causes: First, in Müller et al. [21] the OAS was filled-out by patients’ psychotherapists, who had psychological knowledge and were familiar with psychometric instruments. Observers in the present study were presumably unacquainted with psychological theory and assessment. They might also have responded in a socially desirable way. Second, Müller et al. [21] used multiple observer ratings with the OAS, allowing a more reliable assessment. Third, convergence of TAS-20 and OAS scores was previously only provided for inpatient samples [36]; results might thus not generalize to community samples. A selection bias is unlikely to have influenced present results, as observer- and not-observer rated subjects did not differ significantly in their TAS-20 scores.

### Strengths and limitations

A major strength of the present study lies in the accurately designed priming task as well as in the use of carefully selected and validated stimulus material. Furthermore, our sample was larger in comparison to prior studies and showed a wider range with regard to age and education. Being not confined to a student sample, obtained results appear therefore more generalizable.

However, self-reported alexithymia was in the present sample lower than reported for the German general population in prior studies [27]. This may have impacted results, especially considering the weak correlations between TAS-20 scores with facilitation and inhibition scores. Prior screening of participants with regard to the selection of high and low alexithymics may be beneficial for future studies.

## Conclusions

The present results suggest that abnormalities in information processing in alexithymia might be associated with illness-related information, rather than affective information in general, at least with regard to the processing on an automatic level. Our findings require independent replication to evaluate their robustness, especially with regards to the observed effect of alexithymia on facilitation scores. Future studies ought to distinguish illness-related symptoms from symptoms that can be bodily sensations of emotional arousal as well as signs of illness. This may further highlight whether there are alexithymia-specific differences in processing and attention allocation.
